# Impact of Knowledge Access on Risky Sexual Behaviors Among Chinese Youths to Improve HIV Prevention: Cross-Sectional Study

**DOI:** 10.2196/68339

**Published:** 2025-08-29

**Authors:** Yun Zhang, Jie Jin, Feng Cheng, Xingliang Zhang, Junfang Xu

**Affiliations:** 1 The Second Affiliated Hospital, School of Public Health, Zhejiang University School of Medicine, Number 866, Yuhangtang Road, Hangzhou, 310058, China, 86 18801230482; 2 Hangzhou Center for Disease Control and Prevention (Hangzhou Health Supervision Institution), Hangzhou, China; 3 Vanke School of Public Health, Tsinghua University, Beijing, China; 4 Institute for Healthy China, Tsinghua University, Beijing, China

**Keywords:** Chinese young student, knowledge access, risky sexual behavior, HIV knowledge, health communication, cross-sectional study

## Abstract

**Background:**

HIV infection significantly challenges Chinese youth, with most infections resulting from risky sexual behaviors. Health communication is critical in preventing risky behaviors. Different HIV-related information sources exhibit unique characteristics, affecting young adults’ perception of HIV and sexual behaviors.

**Objective:**

This study aimed to examine the relationship between knowledge accesses and risky behaviors, analyzing the mediating effects of HIV-related knowledge to provide evidence for more effective HIV knowledge dissemination and the reduction of risky behaviors among youth.

**Methods:**

A questionnaire on HIV-related knowledge and behavior of young students was used for the online survey. Knowledge accesses included school education, mass communication, and interpersonal communication. HIV-related knowledge was categorized into fundamental and behavioral guidance knowledge. Risky behavior was defined as multiple sexual partners or unprotected sex. Binary logistic regression was used to analyze the relationship between knowledge accesses, HIV-related knowledge, and risky behaviors. Multiple mediation models constructed by the Process macro (version 4.1) were conducted to determine whether the knowledge level mediated the relationship between knowledge accesses and risky behaviors.

**Results:**

Totally 20,602 respondents participated, with 9541 (46.31%) males and 11,061 (53.69%) females, averaging 20.14 (SD 1.91) years. Mass communication reached 19,030 (92.37%) students, school education reached 17,949 (87.12%), and 11,274 (54.72%) accessed interpersonal communication. Among the 2423 sexually active students, 363 (14.98%) had multiple sexual partners, and 830 (34.26%) engaged in unprotected sex. School education (odds ratio [OR] 0.60, 95% CI 0.46 to 0.78), mass communication (OR 0.64, 95% CI 0.47 to 0.87) and behavioral guidance knowledge (OR 0.79, 95% CI 0.69 to 0.90) were negatively associated with multiple sexual partners, while interpersonal communication (OR 1.41, 95% CI 1.13 to 1.75) was positively associated. School education (OR 0.75, 95% CI 0.62 to 0.91) and behavioral guidance knowledge (OR 0.81, 95% CI 0.73 to 0.89) were negatively correlated with unprotected sexual behaviors, while interpersonal communication (OR 1.27, 95% CI 1.09 to 1.46) and fundamental knowledge (OR 1.17, 95% CI 1.08 to 1.28) were significantly positively correlated. Knowledge accesses indirectly influenced risky behaviors through fundamental and behavioral guidance knowledge. School education had opposing indirect effects of −0.02 and 0.02 through behavioral guidance and fundamental knowledge, mass communication showed effects of −0.04 and 0.01, while interpersonal communication demonstrated indirect effects of 0.01 and 0.01 for both pathways.

**Conclusions:**

Students acquiring HIV knowledge from school education and mass media are less likely to engage in risky behaviors than those learning through interpersonal communication. We should fully leverage the advantages of school education and mass communication, and an emphasis should be placed on behavioral guidance knowledge to promote changes in risky behavior. Knowledge access primarily influences risky behaviors by shaping cultural values and behavioral norms, rather than transmitting information.

## Introduction

HIV infection presents a significant public health challenge due to its high fatality rate and widespread transmission worldwide. Until 2023, a cumulative 39.9 million HIV positive cases, including 1.3 million new infections, were estimated according to the United Nations Program on HIV/AIDS [1]. While China’s concerted efforts have maintained overall HIV prevalence at a low level (approximately <0.1%), there is an alarming rise in infections among young students [2]. Worryingly, nearly 3000 new cases of HIV infection are reported among young adults aged 15 to 24 years every year, increasing at an annual rate of 30%‐50% [3,4]. Therefore, college students in China have become a high-risk group for HIV infection. Sexual transmission is the main mode of HIV spread among young students. Being sexually active and open to casual sex, without correct sexual behavior guidance, young students are prone to have risky behaviors (such as having multiple sexual partners and not using condoms), thereby increasing the risk of HIV infection. Given that, transforming risky behaviors is considered a preventive and control measure for restricting the spread of the HIV pandemic.

Health communication is found to be critical in behavioral intervention [5]. To reduce HIV infection among young students effectively, China conducted HIV health education for students in its 6 major projects aimed at curbing AIDS in 2019 [6]. The public can acquire HIV-related information through various knowledge access points, which can be commonly categorized into organizational communication, mass communication, interpersonal communication, and other types of communication studies [7-9]. Information access exposure exerts an unstoppable and subtle influence on individuals’ cognition and behaviors. Media Richness Theory, proposed by Daft and Lengel [10], suggests that differences in information accesses result in variations in the ability to transmit information. Different HIV-related information sources assign different labels to the nature of the information that affects young adults’ understanding and perception of HIV, ultimately shaping their sexual behaviors. Therefore, the association between different knowledge accesses, knowledge levels, and behaviors deserves attention, as it is essential for identifying effective methods to disseminate HIV-related knowledge and reduce risky behaviors.

However, research on sources of HIV information primarily focuses on the correlation between information sources and HIV-related knowledge [11-13], which fails to fully capture the process of transforming into sexual behavior. Students adopt safe sexual behavior and reject risky behavior based on their accumulation of knowledge, which is considered the ultimate goal of HIV knowledge transmission. Only then can the risk of HIV infection among students be truly reduced [14]. Few studies have explored the relationship between communication channels and sexual behaviors, but they have often neglected to organize these channels [15,16]. Given the diversity of information sources, examining each one individually risks offering incomplete perspectives. Instead of focusing on each specific communication channel, such as distributing leaflets, watching television, or chatting with classmates, it is essential to classify these different channels, which enables the identification of commonalities and differences among them, clarifying their respective strengths and limitations in the dissemination of information.

With the aim of seeking effective information accesses, providing evidence for disseminating knowledge, and reducing risky behavior among young students, this study investigated the knowledge access, knowledge level, and risky behaviors of students, focusing on whether knowledge accesses are associated with sexual behaviors, determining if the HIV-related knowledge mediates the relationship between knowledge accesses and risky behaviors.

## Methods

### Participants

A nonprobability convenience sampling approach was used to recruit young students from 5 colleges located in Zhejiang province, covering comprehensive education, science and technology, medicine, and vocational training, which could represent different types of higher education institutions in Zhejiang province. The sampling strategy aimed to capture the diversity of college students, ensuring a robust representation of this study’s population. The sample size was estimated with the following sample size formula.


$$N=\frac{Z^{2}\times p\;\times \left(1-p\right)}{E^{2}}$$

Z=1.96 (95% confidence level); *P*=.5 (maximum heterogeneity assumption), d=0.05 (5% sampling error), and a response rate of 75% was considered. Given the inherent selection bias of convenience sampling, the minimum calculated sample size was inflated by 30%. As a result, 669 students were required. Finally, 20,602 was investigated and incorporated in the analysis with a strong statistical power.

### Recruitment

The survey was conducted on SoJump (Changsha Ranxing Information Technology Co, Ltd), a widely used Chinese online platform with a user-friendly interface and broad applicability in academic research [17]. The SoJump platform uses cookies to prevent duplicate submissions, and we set a mandatory requirement that participants complete all survey items before submission to ensure data integrity. Participants were recruited voluntarily through QR codes distributed by school counselors via WeChat (Tencent Holdings Limited) and QQ (Tencent Holdings Limited) groups, ensuring accessibility across diverse student populations. Data collection took place from November 2022 to June 2023. The inclusion criteria of the college students were (1) registered students of higher education institutions during the survey period and (2) willing to participate in the survey.

### Variable Measurement

A questionnaire on HIV/AIDS knowledge and behavior among young college students was used to conduct the online survey. The content of the questionnaire included demographic characteristics (age, gender, major, etc), knowledge accesses, HIV-related knowledge, and sexual behaviors. Knowledge accesses in our study included school education, mass communication, and interpersonal communication, which are always discussed in educational communication [18]. As an educational organization, a school can disseminate HIV-related knowledge by organizing classes and giving lectures, which play an important role in the education of young students. Mass communication means the way to transmit information to the public via mass media, such as the internet, radio, television, books, newspapers, and so on. Interpersonal communication refers to direct interaction between 2 or more people to exchange information, such as acquiring HIV-related knowledge from parents, classmates, or friends.

A commonly used scale, “8 questions for youths,” was used to measure HIV-related knowledge [19,20], which refers to the 8 items of HIV/AIDS core knowledge proposed by the Chinese Center for Disease Control and Prevention in 2016 [21]. To better understand the roles of different types of knowledge in cognitive construction and behavioral intervention, HIV-related knowledge was classified into 2 major categories: fundamental knowledge and behavioral guidance knowledge. The classification aligns with the framework of the “Core Information on HIV/AIDS Prevention Education for Young Students (2021)” issued by the China Center for Disease Control and Prevention [22], which originally divides HIV knowledge into 4 modules: awareness of harm, legal regulations, preventive measures, and testing and treatment. However, to ensure measurement reliability and stability, dimension grouping must consider item correlations and internal consistency. With only 8 items, subdividing into 4 modules risks low reliability and modeling challenges. Therefore, the first 2 modules (awareness of harm, legal regulations) were merged into fundamental knowledge to establish a basic understanding of HIV/AIDS and its societal impacts, such as “HIV/AIDS is a serious, incurable infectious disease” and “national laws protect the rights of people living with HIV, including marriage, employment, and education.” The latter 2 modules (preventive measures, testing, and treatment) were combined into behavioral guidance knowledge, focusing on specific preventive actions and behavioral recommendations, such as “daily life and learning contact do not transmit HIV” and “seeking HIV testing and counseling after risky behaviors is essential.”

Risky behavior in our study was defined as having multiple sexual partners or engaging in unprotected sexual behaviors. Having more than 2 sexual partners was considered as having multiple sexual partners. Not using a condom every time during sex was considered unprotected sexual behavior, while consistent condom use was regarded as never engaging in unprotected sex.

### Statistical Analysis

Data analysis was conducted using SPSS (version 19.0; IBM Corp). Chi-square test was used to analyze the differences in knowledge acquisition methods among people with different characteristics. Taking multiple sexual partners (no multiple sexual partners=0 and had multiple sexual partners=1) and unprotected sexual behavior (no unprotected sexual behavior=0 and had unprotected sexual behaviors=1) as dependent variables. The 3 forms of knowledge accesses, including school education, mass communication, and interpersonal communication, and HIV-related knowledge (containing fundamental knowledge and behavioral guidance knowledge of HIV), were used as the core independent variables. The effects of knowledge access and HIV-related knowledge on sexual behaviors were analyzed using binary logistic regression. Specifically, confounding factors such as gender, age, major, and grade were incorporated as covariates in the logistic regression models to adjust for their effects.

Process macro (version 4.1, model 4) [23] program compiled by Hayes [24] was used to construct multiple mediation models of “knowledge accesses-knowledge-risky behaviors” to explore how different knowledge accesses (school education, mass communication, and interpersonal communication) directly affect risky behaviors and how they indirectly affect risky behaviors through fundamental knowledge and behavioral guidance knowledge. Multiple mediation (model 4) analysis was based on bootstrap, with 5000 repeated sampling times. The confounding factors, such as gender, age, major, and grade, were controlled for analysis. The significance test was 2-sided, and the level of significance was set at *P*<.05.

### Ethical Considerations

This study’s protocol and informed consent procedures were approved by the Ethics Review Committee of the School of Public Health, Zhejiang University (ZGL202306-9). Before filling out the questionnaire, participants were informed about the purpose, process, potential risks, and benefits of the study, and it was voluntary to withdraw or end the investigation. No personally identifiable information was collected. Participants were provided with nonmonetary incentives, including free access to HIV/AIDS risk assessment tools and health consultation services.

## Results

### Sample Characteristics

The study population comprised 20,602 students. There were 9541 (46.31%) males and 11,061 (53.69%) females. The average age was 20.14 (SD 1.91) years. The number of freshmen, sophomores, juniors, and seniors was 10,339 (50.18%), 4679 (22.71%), 2532 (12.29%), and 974 (4.73%), respectively. A total of 2078 (10.09%) were graduate students. In terms of major, 7835 (38.03%) were majoring in medicine, 6729 (32.66%) were enrolled in science and technology programs, 4947 (24.01%) were studying literature, arts, or management, and 1091 (5.30%) were in vocational education. A total of 2423 students had sex. Among them, 363 (14.98%) had multiple sexual partners, and 830 (34.26%) engaged in unprotected sexual behaviors.

### HIV-Related Knowledge and Knowledge Accesses

The average HIV-related knowledge score of 20,602 college students was 6.95 (SD 1.32). A total of 8485 (41.19%) students answered all questions correctly. Further, 125 (0.61%) students scored 0. The average score of fundamental knowledge was 3.26 (SD 0.88), and the average score of behavioral guidance knowledge was 3.69 (SD 0.69; Table 1).

**Table 1.** Distribution of responses to HIV-related knowledge among college students in China, a cross-sectional online survey in 2022‐2023.

HIV-related knowledge	Correct count	Accuracy (%)
Fundamental knowledge		
AIDS is a serious and incurable infectious disease.	15,623	75.83
The main transmission mode of AIDS among young students in China is male homosexual, followed by heterosexual behavior.	15,860	76.98
A person infected with HIV can be identified by their appearance.	17,918	86.97
The rights of people living with HIV, including marriage, employment, and education, are protected by national law.	17,669	85.76
Behavioral guidance knowledge		
Can daily life and learning contact infect you with HIV?	18,499	89.79
Consistent and correct condom use reduces the risk of acquiring and transmitting HIV.	19,671	95.48
The use of new psychoactive substances increases the risk of HIV infection.	17,985	87.30
Seeking HIV testing and counseling after engaging in risky behaviors is essential.	19,894	96.56

The audience for mass communication included 19,030 (92.37%) students; 17,949 (87.12%) students received school education to acquire HIV-related knowledge; 11,274 (54.72%) obtained HIV-related knowledge via interpersonal communication, accounting for a relatively lower proportion. Further, 9645 (46.82%) students used all 3 forms of information channels to obtain knowledge, whereas only 2596 (12.60%) students relied on a single source for obtaining knowledge (Multimedia Appendix 1).

### Distribution Characteristics of Knowledge Accesses of Young Students

Students with different genders, grades, ages, majors, and sexual behaviors exhibited varying preferences for information sources. Most of those relying on interpersonal communication were male (*χ*²_1_=4.19, *P*=.04). In terms of age, students under 20 primarily acquired knowledge through school education (*χ*²_1_=176.04, *P*<.01) and interpersonal communication (*χ*²_1_=82.67, *P*<.01), and the audience of mass communication (*χ*²_1_=25.48, *P*<.01) was mainly composed of students aged 20 years and older. Medical students were the main audience for both school education (χ²_3_=160.17, *P*<.01) and mass communication (*χ*²_3_=54.22, *P*<.01), whereas vocational students mostly preferred interpersonal communication (*χ*²_3_=58.72, *P*<.01). Students who never had sexual behavior mainly chose school education (*χ*²_1_=80.53, *P*<.01) and mass communication (*χ*²_1_=4.19, *P*=.04). In terms of risky sexual behaviors, those with multiple sexual partners tended to obtain HIV-related knowledge through interpersonal communication (*χ*²_1_=4.69, *P*=.03; Table 2).

**Table 2.** Chi-square test of HIV-related knowledge acquisition approaches among young students in China, a cross-sectional online survey in 2022‐2023.

Items	Number	School education	Mass communication	Interpersonal communication
Gender				
Male	9541	8121 (85.12)^a^	8555 (89.67)^a^	5294 (55.49)^a^
Female	11,061	9828 (88.85)^a^	10,475 (94.70)^a^	5980 (54.06)^a^
*χ*² *(df)*		63.72 (1)	184.36 (1)	4.19 (1)
*P*		<.01	<.01	.04
Age (years)				
≤20	13,835	12,353 (89.29)^a^	12,689 (91.72)^a^	7876 (56.93)^a^
>20	6767	5596 (82.70)^a^	6341 (93.70)^a^	3398 (50.21)^a^
*χ*² *(df)*		176.04 (1)	25.48 (1)	82.67 (1)
*P*		<.01	<.01	<.01
Major				
Medicine	7835	7098 (90.59)^a^	7301 (93.18)^a^	4521 (57.70)^a^
Science and technology	6729	5650 (83.96)^a^	6193 (92.03)^a^	3541 (52.62)^a^
Literature, arts, or management	4947	4228 (85.47)^a^	4587 (92.72)^a^	2578 (52.11)^a^
Vocational education	1091	973 (89.18)^a^	949 (86.98)^a^	634 (58.11)^a^
*χ*² *(df)*		160.17 (3)	54.22 (3)	58.72 (3)
*P*		<.01	<.01	<.01
Grade				
Freshman	10,339	9289 (89.84)^a^	9421 (91.12)^a^	5945 (57.5)^a^
Sophomore	4679	4152 (88.74)^a^	4344 (92.84)^a^	2625 (56.1)^a^
Junior	2532	2107 (83.21)^a^	2395 (94.59)^a^	1309 (51.70)^a^
Senior	974	807 (82.85)^a^	907 (93.12)^a^	499 (51.23)^a^
Graduate student	2078	1594 (76.71)^a^	1963 (94.47)^a^	896 (43.12)^a^
*χ*² *(df)*		330.30 (4)	55.78 (4)	162.87 (4)
*P*		<.01	<.01	<.01
Having sexual intercourse?				
Yes	2423	1972 (81.39)^a^	2213 (91.33)^a^	1358 (56.05)^a^
No	18,179	15,977 (87.89)^a^	16,817 (92.51)^a^	9916 (54.55)^a^
*χ*² *(df)*		80.53 (1)	4.19 (1)	1.94 (1)
*P*		<.01	.04	.16
Having multiple sexual partners?				
Yes	363	281 (77.41)^a^	313 (86.23)^a^	219 (60.33)^a^
No	20,239	17,668 (87.3)^a^	18,717 (92.48)^a^	11,055 (54.62)^a^
*χ*² *(df)*		31.07 (1)	19.79 (1)	4.69 (1)
*P*		<.01	<.01	.03
Having unprotected sex?				
Yes	830	673 (81.08)^a^	747 (90.00)^a^	478 (57.59)^a^
No	19,772	17,276 (87.38)^a^	18,283 (92.47)^a^	10,796 (54.60)^a^
*χ*² *(df)*		28.11 (1)	6.89 (1)	2.87 (1)
*P*		<.01	<.01	.09

a n (%).

### Association Between Knowledge Accesses, HIV-Related Knowledge, and Multiple Sexual Partner Behaviors

According to multivariate logistic regression analysis, compared with students who did not acquire knowledge through school education and mass media, those who acquired knowledge through school education (odds ratio [OR] 0.60, 95% CI 0.46 to 0.78) and mass media (OR 0.64, 95% CI 0.47 to 0.87) were less likely to have multiple sexual partners. Students who acquired knowledge through interpersonal communication (OR 1.41, 95% CI 1.13 to 1.75) were more likely to have multiple sexual partners than those who did not. A higher level of behavioral guidance knowledge (OR 0.79, 95% CI 0.69 to 0.90) was associated with a lower likelihood of having multiple sexual partners; the level of fundamental knowledge had no significant effect on the behavior of having multiple sexual partners (Table 3).

**Table 3.** Association between knowledge accesses, HIV-related knowledge, and multiple sexual partner behaviors among Chinese college students^a^.

Variables	B	SE	Wald	*P* value	OR (95% Cl)
Knowledge accesses					
School education	−0.51	0.13	14.94	<.01	0.60 (0.46, 0.78)
Mass communication	−0.45	0.16	8.05	<.01	0.64 (0.47, 0.87)
Interpersonal communication	0.34	0.11	9.33	<.01	1.41 (1.13, 1.75)
HIV-related knowledge					
Fundamental knowledge	0.11	0.06	2.69	.10	1.11 (0.98, 1.26)
Behavioral guidance knowledge	−0.24	0.07	12.53	<.01	0.79 (0.69, 0.90)

a Logistic regression analysis was conducted to examine the relationship between having multiple sexual partners (independent variable) and knowledge accesses and levels (dependent variables), controlling for gender, age, major, and grade. Hosmer-Lemeshow test *P*>.5.

### Association Between Knowledge Accesses, HIV-Related Knowledge, and Unprotected Sexual Behaviors

The regression results showed that students who acquired knowledge through school education (OR 0.75, 95% CI 0.62 to 0.91) were less likely to engage in unprotected sexual behaviors than those who did not; students who obtained knowledge through interpersonal communication (OR 1.27, 95% CI 1.09 to 1.46) were more likely to engage in unprotected sexual behaviors. There was no significant association between mass media exposure and unprotected sex behaviors. Fundamental knowledge (OR 1.17, 95% CI 1.08 to 1.28) was associated with an increased likelihood of engaging in unprotected sexual behaviors. Behavioral guidance knowledge (OR 0.81, 95% CI 0.73 to 0.89) was negatively correlated with unprotected sexual behaviors (Table 4).

**Table 4.** Association of knowledge accesses, HIV-related knowledge, and unprotected sexual behaviors among Chinese college students.^a^

Variables	B	SE	Wald	*P* value	OR (95% Cl)
Knowledge accesses					
School education	−0.29	0.1	8.95	<.01	0.75 (0.62, 0.91)
Mass communication	−0.19	0.12	2.44	.12	0.83 (0.65, 1.05)
Interpersonal communication	0.24	0.07	9.96	<.01	1.27 (1.09, 1.46)
HIV-related knowledge					
Fundamental knowledge	0.16	0.05	12.73	<.01	1.17 (1.08, 1.28)
Behavioral guidance knowledge	−0.21	0.05	18.47	<.01	0.81 (0.73, 0.89)

a Logistic regression analysis was conducted to examine the relationship between having unprotected sex (independent variable) and knowledge accesses and levels (dependent variables), controlling for gender, age, major, and grade. Hosmer-Lemeshow test *P*>.5.

### Mediating Effect of HIV-Related Knowledge on the Correlation Between Knowledge Accesses and Risky Behaviors

The results of multiple mediation models showed that both fundamental knowledge and behavioral guidance knowledge of HIV acted as joint mediators in the relationship between various knowledge accesses (school education, mass communication, and interpersonal communication) and risky behaviors.

**Figure 1.**
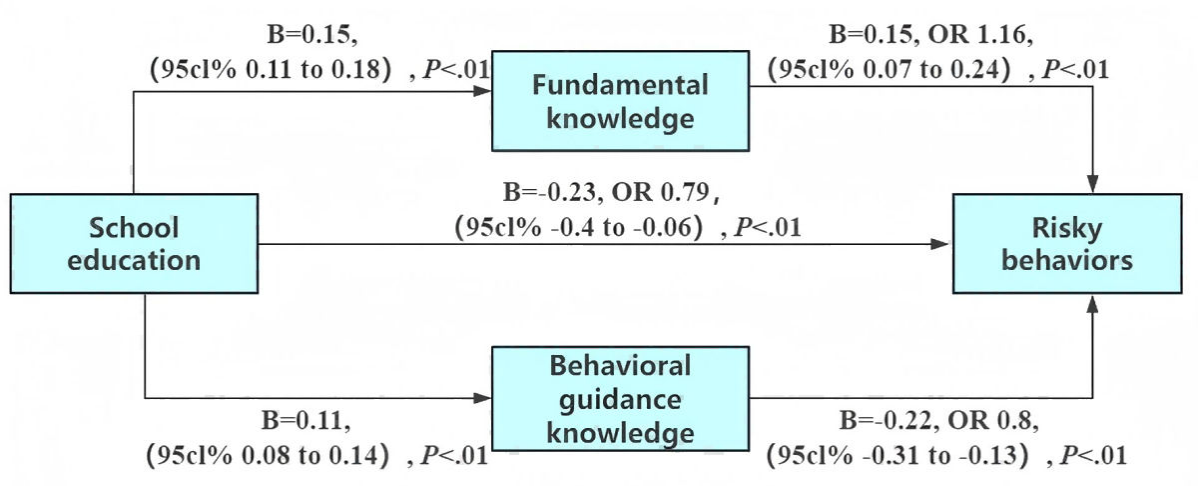
Mediation model illustrating the impact of school education on risky behaviors among Chinese students via fundamental knowledge and behavioral guidance knowledge. Path diagram illustrating the dual mediation mechanism through fundamental knowledge (**M1**) and behavioral guidance knowledge (**M2**) between school education (**X**) and risky behaviors (**Y**). Values on the arrows represent unstandardized regression coefficients (**B**). ORs and their 95% CIs are also reported. The model controlled for gender, age, major, and grade. OR: odds ratio.

The regression results indicated that school education was positively associated with behavioral guidance knowledge (B=0.11, *P*<.01) and fundamental knowledge (B=0.15, *P*<.01). When simultaneously predicting risky behaviors with school education, behavioral guidance knowledge, and fundamental knowledge, both school education (B=−0.23, OR =0.79, 95% CI −0.40 to −0.06, *P*<.01) and behavioral guidance knowledge (B=−0.22, OR 0.8, 95% CI −0.31 to −0.13, *P*<.01) were negatively associated with risky behaviors, while fundamental knowledge was positively associated with risky behaviors (B=0.15, OR 1.16*,* 95% CI 0.07 to 0.24, *P*<.01; Figure 1). The indirect effects of behavioral guidance knowledge and fundamental knowledge were −0.02 and 0.02, respectively. The total indirect effect was nonsignificant, suggesting that the opposing effects of the 2 mediating pathways may offset each other (Table S3 in Multimedia Appendix 2).

**Figure 2.**
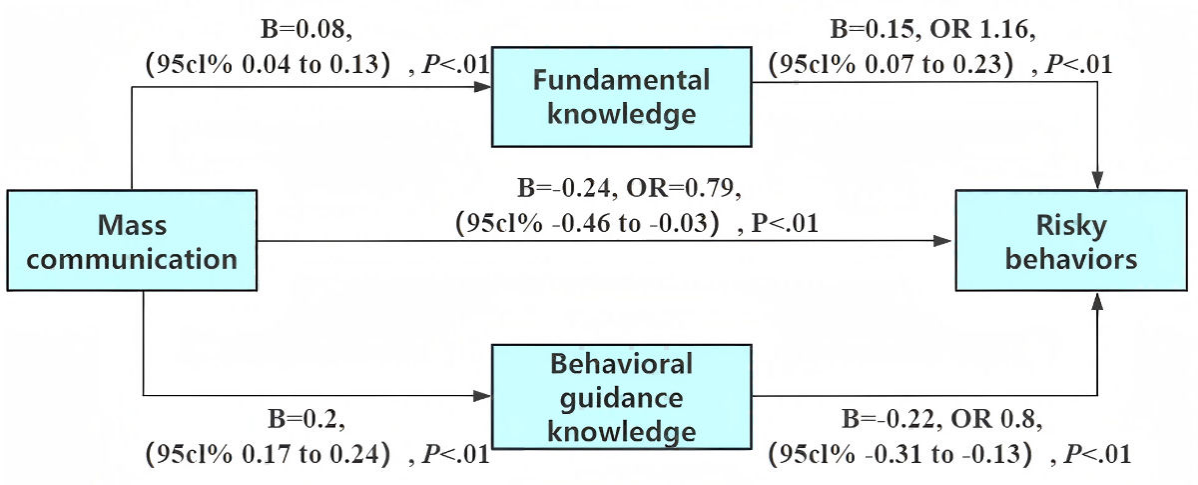
Mediation model illustrating the impact of mass communication on risky behaviors among Chinese students via fundamental knowledge and behavioral guidance knowledge. Path diagram illustrating the dual mediation mechanism through fundamental knowledge (**M1**) and behavioral guidance knowledge (**M2**) between mass communication (**X**) and risky behaviors (**Y**). Values on the arrows represent unstandardized regression coefficients (**B**). ORs and their 95% CIs are also reported. The model controlled for gender, age, major, and grade. OR: odds ratio.

Mass communication was positively associated with behavioral guidance knowledge (B=0.2, *P*<.01) and fundamental knowledge (B=0.08, *P*<.01). When predicting risky behaviors with mass communication, behavioral guidance knowledge, and fundamental knowledge, both mass communication (B=−0.24, OR 0.79, 95% CI −0.46 to −0.03, *P*<.01) and behavioral guidance knowledge (B=−0.22, OR 0.8, 95% CI −0.31 to −0.13, *P*<.01) were negatively associated with risky behaviors, while fundamental knowledge was positively associated (B=0.15, OR 1.16, 95% CI 0.07 to 0.23, *P*<.01; Figure 2). The indirect effects of behavioral guidance knowledge and fundamental knowledge were −0.04 and 0.01, respectively (Table S5 in Multimedia Appendix 2).

**Figure 3.**
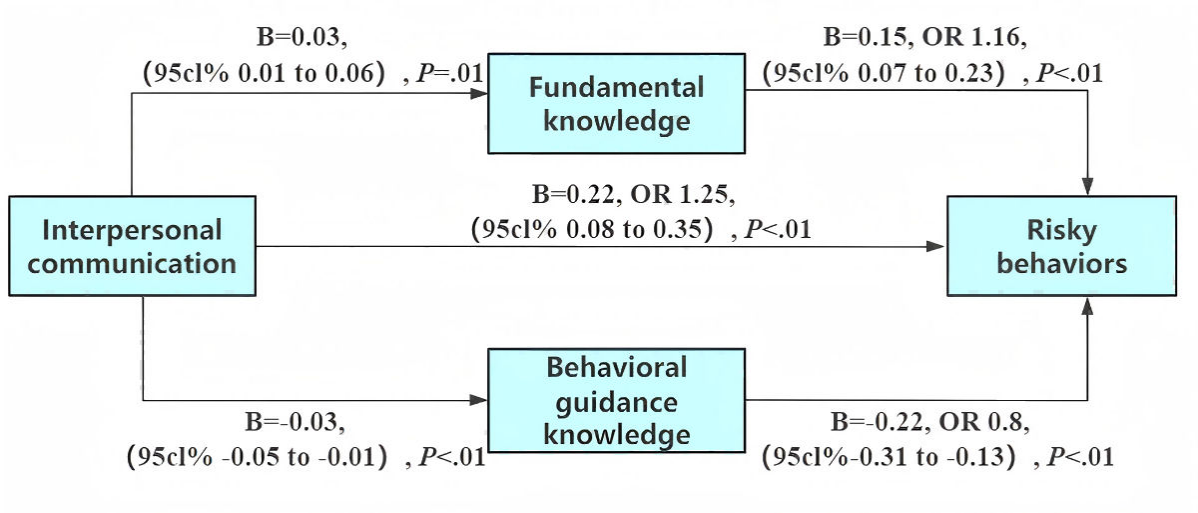
Mediation model illustrating the impact of interpersonal communication on risky behaviors among Chinese students via fundamental knowledge and behavioral guidance knowledge. Path diagram illustrating the dual mediation mechanism through fundamental knowledge (**M1**) and behavioral guidance knowledge (**M2**) between interpersonal communication (**X**) and risky behaviors (**Y**). Values on the arrows represent unstandardized regression coefficients (**B**). ORs and their 95% CIs are also reported. The model controlled for gender, age, major, and grade. OR: odds ratio.

Interpersonal communication was negatively associated with behavioral guidance knowledge (B=−0.03, *P*<.01) and positively associated with fundamental knowledge (B=0.03, *P*<.01). Interpersonal communication showed a direct positive association with risky behaviors (B=0.22, OR 1.25, 95% CI 0.08 to 0.35, *P*<.01), while both behavioral guidance knowledge (B=−0.22, OR 0.8, 95% CI −0.31 to −0.13, *P*<.01) and fundamental knowledge (B=0.15, OR 1.16, 95% CI 0.07 to 0.23, *P*<.01) demonstrated significant effects on risky behaviors. Interpersonal communication directly had a positive impact on risky behaviors and increased risky behaviors by improving fundamental knowledge and reducing behavioral guidance knowledge (Figure 3). The indirect effects of behavioral guidance knowledge and fundamental knowledge were 0.01 and 0.01, respectively (Table S7 in Multimedia Appendix 2).

## Discussion

### Principal Findings

Based on the investigation of 20,602 Chinese university students, this cross-sectional study revealed distinct associations between HIV knowledge, access channels, and risky sexual behaviors (unprotected sex and multiple partners). While school education and mass media access were associated with reduced risky sexual behaviors, interpersonal communication correlated with increased risk. What is more, higher HIV behavioral guidance knowledge was linked to safer behaviors, whereas fundamental knowledge paradoxically was associated with more risky behaviors. Mediation analysis indicated that the direct influence of knowledge accesses, likely via shaping norms, was more dominant than their indirect effects through knowledge transmission.

A score of 6 or more is considered to be up to the standard for HIV health education knowledge [22,25]. In this survey, 89.80% (18,501/20,602) of the students answered more than 6 questions correctly. There is still a long way to go to meet the goal of over 95% of young students being aware of HIV, as outlined in the Implementation Plan for Curbing the Spread of HIV/AIDS (2019‐2022). College students mainly obtain HIV-related information through mass media and school education, which aligns with previous studies conducted in China [26-28]. This may be attributed to the rapid development of the internet, allowing young adults to easily obtain various information about HIV/AIDS through search engines, social media, and other platforms [29,30]. School, being central to students’ daily lives, offers a structured environment conducive to HIV educational campaigns, which has the advantage of centralized publicity. Therefore, mass communication and school education are always favored by students. However, fewer students reported acquiring HIV-related knowledge through interpersonal communication, which may be affected by Chinese traditional culture. Sexual content is often regarded as an obscure and sensitive topic, and parents or friends will deliberately avoid such topics. Similar patterns, rooted in shared East Asian cultural influences, which are culturally conservative and emphasize collectivism, have been observed in studies on Korean youths, who also primarily obtain HIV knowledge from mass media and school, rather than interpersonal channels [31]. However, a substantial body of Western literature documents the importance of parent-child communication and peer interaction in preventing risky sexual behaviors [32-34]. Cultural values can influence the sources of HIV information sought by young people, with collectivist and conservative cultures potentially prioritizing indirect or institutional channels over direct, potentially confrontational, and interpersonal dialogues.

Access to HIV-related knowledge through school education and mass media may have a positive association with a reduction in behaviors such as having multiple sexual partners and engaging in unprotected sex, as indicated by our regression analysis. As a vital form of organizational communication, school education holds a high level of authority and credibility, which is embodied in the strict censorship system of knowledge dissemination, so young students are inclined to trust the information they receive in school. Additionally, organizational communication may be mandatory. For instance, schools can introduce compulsory HIV education courses or make it mandatory for students to attend relevant lectures, which can break through the personal “information cocoons” to some extent, where students might otherwise only seek information aligned with their existing interests or biases. As a result, students are exposed to more comprehensive and accurate HIV-related knowledge than they might seek out on their own. The mass media, including newspapers, radio, television, and the internet, also have unique advantages. Mass media is characterized by its speed of transmission and the breadth of its content, allowing it to rapidly achieve widespread information coverage. The ability of mass media to disseminate knowledge in engaging and entertaining formats helps overcome the discomfort associated with “face-to-face” discussions on sensitive topics, making it easier for students to accept and retain the information.

The association between school education and risky behaviors is stronger than that of mass media, which is consistent with the research of Chinese scholars [35]. Unlike online platforms, where information can be overwhelming and fragmented, school curricula are designed to be comprehensive and progressive, allowing students to build their understanding step by step. Additionally, the accuracy of information from mass media is often difficult to guarantee. While the internet has become the primary medium through which young students access information, its less regulated nature requires students to exercise caution. The sheer volume of content, along with the prevalence of unreliable or false information, underscores the importance of seeking official and authoritative channels to acquire accurate HIV knowledge. This preference for structured and authoritative sources, along with their effectiveness, is deeply rooted in China’s collectivist culture [36], particularly its emphasis on institutional trust, hierarchical order, and the role of established authority in knowledge dissemination. As state-sanctioned institutions, schools provide a reliable and systematic avenue for addressing sensitive topics such as sexual health. Culturally, students are conditioned to view knowledge imparted by teachers and formal curricula as vetted and trustworthy.

Mass communication did not have a significant effect on unprotected sex, but did have a significant effect on the reduction of multiple sexual partners. When disseminating information, mass media always tailor content and format to align with the public’s interests and concerns. Compared to topics such as “correct condom use during sex,” the issue of multiple sexual partners is much more provocative and sensitive, which can arouse the curiosity of college students. It is suggested that mass media should not only consider the attractiveness of the content, but also shoulder the social responsibility of providing more comprehensive coverage of HIV/AIDS prevention.

By regression analysis, it was found that students who acquired knowledge through interpersonal communication were more likely to have multiple sexual partners and engage in unprotected sex. This may be due to the lack of clarity or comprehensiveness in the information disseminated through personal networks, which undermines effective risk perception and the adoption of protective behaviors. In the mediation analysis, we found that interpersonal communication negatively predicted behavioral guidance knowledge, which may be able to support this view. First, HIV-related knowledge acquired from family members or friends is relatively superficial and unsystematic [29]. Knowledge transmitted may be based on personal understanding, hearsay, or limited media information, making it difficult to guarantee the accuracy and comprehensiveness. The widespread cultural constraints in East Asian societies have further limited the acquisition of knowledge through interpersonal communication, where sexuality is often considered a sensitive and taboo topic [34,37]. The cultural stigma makes open and direct discussion about sexual health challenging within family and peer circles. Even when discussions occur, the focus tends to be on the moral consequences or general harms of the disease, rather than on practical prevention methods, largely due to discomfort with explicit sexual discourse. Sensitive topics, such as condom use or specific sexual practices, might be perceived as impolite and embarrassing. Family members or friends, constrained by these cultural norms, might default to abstract moral warnings or general admonitions rather than concrete and actionable prevention strategies. In Latino families that emphasize conservative culture, studies have also pointed out that Latinos are less willing to provide children with information about sex and AIDS, telling them to protect themselves, but not giving them specific information on how to do it [38]. While the intention may be to guide young students toward safe sexual behavior, the cultural barriers to open dialogue create a critical gap between the desired outcome and the actual knowledge and skills acquired.

Moreover, the efficacy of knowledge acquisition through interpersonal communication is often undermined by personal filters. Emotional factors frequently mediate the transmission of information, leading to its simplification or distortion. When receiving HIV knowledge from a close friend, the emotional bond might lead to a downplaying of risks. Casual language or humor used to address the topic can create a false sense of security and diminish the perceived seriousness of HIV’s harms. The personal filtering effect is amplified in cultural contexts that prioritize social harmony and discomfort avoidance [39]. Out of a desire to protect the listener from anxiety or maintain normalcy, friends or family members may inadvertently or intentionally simplify or downplay the risks of HIV. Furthermore, within Chinese culture, the concept of filial piety may discourage younger individuals from questioning or challenging potentially inaccurate information from parents or older adults, even if they intuitively perceive discrepancies. It is recommended that young students, especially the main audience group of interpersonal communication, consult HIV curricula in schools or official websites to avoid being misled by false information when discussing HIV with parents, relatives, or friends. When acting as communicators, young students must ensure the accuracy of the information, prioritize the transmission of behavioral guidance knowledge, and treat the topic with appropriate seriousness.

However, this study could not determine whether the acquisition of HIV-related knowledge through interpersonal communication occurs in succession with risky behaviors, so it could not clarify whether interpersonal communication promotes the occurrence of risky sexual behaviors. As mentioned above, sexual topics are inherently highly sensitive and difficult to discuss openly in China. Individuals who proactively engage in interpersonal communication about HIV information are likely to be those who have already engaged in high-risk behaviors, and similar views can be found [40]. Their exchanges are more often based on personal experiences or seeking social support, rather than systematically acquiring scientific prevention knowledge. This tendency to seek social support after engaging in risky behaviors may explain why acquiring knowledge through interpersonal communication is positively correlated with risky sexual behaviors.

According to the “Knowledge, Attitude, and Practice” model, knowledge is the foundation of behavior change, but a high level of HIV knowledge does not always lead to safe behaviors, reflecting the phenomenon of “separation.” This study found that a higher level of fundamental knowledge of HIV may be related to more risky sexual behaviors, while behavioral guidance knowledge plays a positive role in reducing such behaviors. The findings suggest that different types of knowledge have varying effects on risky behaviors. Fundamental knowledge provides a broad and foundational understanding of HIV, but may not effectively translate into safe sexual practices. In fact, a strong grasp of fundamental knowledge may foster a sense of overconfidence or “risk underestimation,” causing students to lower their vigilance during sexual behaviors. Additionally, students may misunderstand the legal protection for HIV-positive individuals, regarding such policies as shelters for risky sexual behaviors. Consequently, fundamental knowledge may be insufficient to reduce risky behaviors alone. Behavioral guidance knowledge is relatively more specific and practical, directly guiding individuals to adopt HIV prevention measures, such as correctly using condoms or knowing when and where to get tested after potential exposure. Unlike fundamental knowledge, which may raise awareness but leave gaps in practical application, behavioral guidance knowledge equips students with concrete tools to lower their risk and facilitates the translation of knowledge into safe behaviors. Therefore, the focus should be on strengthening the dissemination of HIV behavioral guidance knowledge. However, it does not imply that we should reduce the publicity of HIV fundamental knowledge, which serves as the foundation for building a comprehensive understanding of HIV. HIV health education should emphasize behavioral guidance knowledge while constantly reinforcing fundamental knowledge [41], which enables college students to gain a more systematic and comprehensive grasp of HIV information. By combining a solid foundation of fundamental knowledge with a stronger focus on prevention, young students will be better positioned to apply information in real-life situations, significantly lowering their likelihood of engaging in risky sexual behaviors.

Based on the results of the mediation analysis, it was found that school education, mass communication, and interpersonal communication all influence risky behaviors through both behavioral guidance knowledge and fundamental knowledge. However, the overall mediation effects were relatively small, and the influence paths varied across these channels. It suggests that the impact of these knowledge accesses on behavior may not primarily operate through knowledge acquisition, but is instead driven more by its direct influence. Media systems serve not only as tools for knowledge transmission but also as sites for the production and reproduction of cultural capital, influencing individuals by shaping cultural values and behavioral norms. School education’s influence on risky behaviors may arise less from knowledge acquisition and more from the structured and organized environment it provides, where students may feel external pressures or benefit from behavioral norms enforced by the school setting. Interpersonal communication easily fosters an open atmosphere for young students, making students more likely to have a fluke or curiosity, and ultimately leading to a higher likelihood of engaging in risky behaviors. The indirect effect of mass communication on reducing risky behaviors by improving the knowledge level of individuals is relatively strong, especially through the enhancement of behavioral guidance knowledge. It may be due to the voluntary nature of information seeking in mass communication that highlights the importance of knowledge acquisition initiatives. This personalized, needs-driven communication approach is more effective than passive teaching methods in influencing behavior by increasing the level of knowledge.

### Limitations

The study is subject to some limitations. First, while the cross-sectional design revealed associations between knowledge access channels and risky behaviors, our study could not explore the causality of knowledge accesses and risky behaviors. Future research should use longitudinal or quasi-experimental designs to clarify causality. Second, the generalizability of the findings in this study is limited by its scope. The level of economic development and the open environment in Zhejiang province may have led to an overestimation of students’ HIV knowledge and their openness to discussing the topic. The results of this study may also be influenced by cultural context; while Zhejiang province is somewhat representative of broader Chinese values, caution is required when directly applying these findings to all university student populations or to distinctly different cultural contexts, given the inherent geographical and cultural differences. This also underscores the need for future cross-cultural research to further analyze and explore effective approaches to HIV education across diverse cultural backgrounds. Third, by combining the 8 questions for measuring HIV-related knowledge into 2 dimensions, we may not have fully explored the intrinsic connections among the questions. Future research should develop more refined knowledge classification systems and measurement tools, ensuring a sufficient number of items to capture the nuanced effects of different knowledge types on behavior. Moreover, considering the sensitivity of sexual behaviors, the young students may provide social desirability responses, which may influence the results. Finally, for statistical analysis, more demographic variables deserve to be included, such as sexual orientation and family economic status. Interaction effects of variables can provide valuable insights into the conditional relationships between key factors, which future research could focus on.

### Conclusions and Policy Implications

This study highlights that HIV prevention education should strategically combine the strengths of different transmission pathways. Specifically, it should leverage the authority and structured nature of school-based programs while capitalizing on the broad reach of mass media. Guidance should be provided for schools to reform educational content and formats, emphasizing the encouragement of voluntary student participation rather than mandatory intervention. Online public discourse should be monitored, and the government must intervene to correct misinformation that misleads students. To address the potential risks of informal interpersonal communication, it is crucial to enhance its quality. A knowledge certification system for peer educators should be established, and student leaders should be trained to disseminate accurate prevention information. Additionally, interpersonal communication quality should be included in HIV/AIDS prevention assessment metrics. Regarding knowledge structure, we advocate prioritizing actionable, practical HIV/AIDS behavioral guidance knowledge, focusing on risk-reduction skills to bridge the knowledge-behavior gap. Transmission pathways not only convey information but also shape behavioral norms. Schools can use rituals such as anti-AIDS pledge ceremonies to strengthen students’ responsibility, while mass media can reshape perceptions and promote supportive environments for behavior change.

## Supplementary material

10.2196/68339 Multimedia Appendix 1 Distribution of AIDS knowledge acquisition approaches among college students in China, a cross-sectional online survey in 2022-2023.

10.2196/68339 Multimedia Appendix 2 Comprehensive overview of sample characteristics and mediation analysis results.

## Abbreviations

OR: odds ratio
