# Supplementary figures and images for: Impact of Knowledge Access on Risky Sexual Behaviors Among Chinese Youths to Improve HIV Prevention: Cross-Sectional Study
Source: JMIR Public Health Surveill. 2025 Aug 29;11:e68339. doi: 10.2196/68339 (PMC12396772; doi:10.2196/68339)

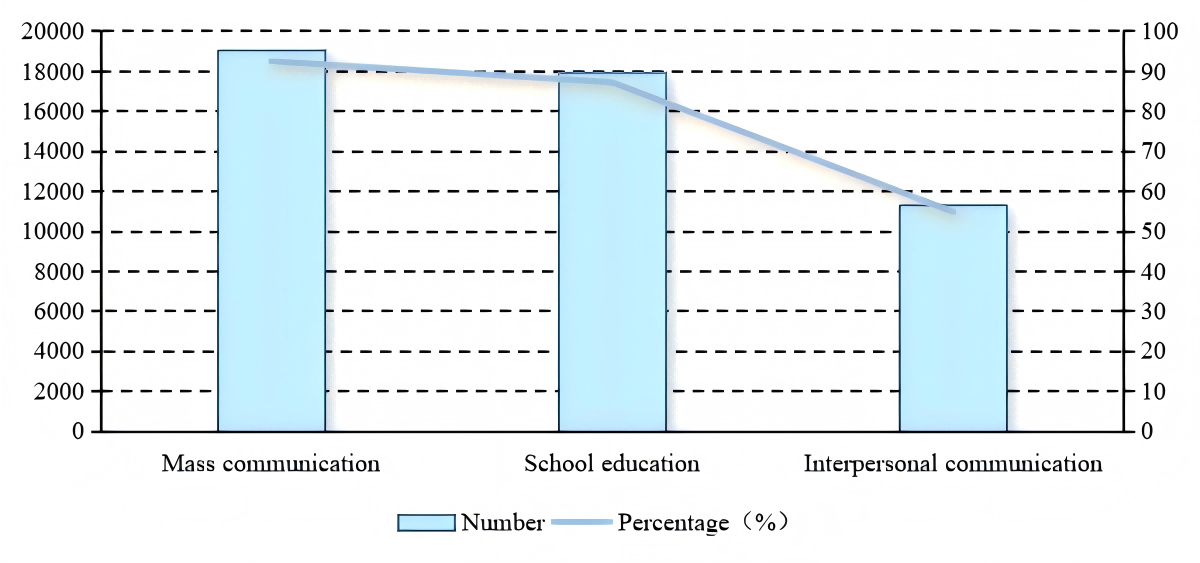

Supplement: Multimedia Appendix 1 — Distribution of AIDS knowledge acquisition approaches among college students in China, a cross-sectional online survey in 2022-2023. [file publichealth-v11-e68339-s001.png]
