# Supplementary material for: Impact of Knowledge Access on Risky Sexual Behaviors Among Chinese Youths to Improve HIV Prevention: Cross-Sectional Study
Source: JMIR Public Health Surveill. 2025 Aug 29;11:e68339. doi: 10.2196/68339 (PMC12396772; doi:10.2196/68339)
Supplement: Multimedia Appendix 2 — Comprehensive overview of sample characteristics and mediation analysis results. [file publichealth-v11-e68339-s002.docx]

**Table S1.** Descriptive statistics of sample characteristics and key variables (N = 20602).

| **Items** | **Category** | **Number** | **Percentage (%)** |
| --- | --- | --- | --- |
|  |  |  |  |
| **Demographic Information** |  |  |  |
| Gender | Male | 9541 | 46.31 |
|  | Female | 11061 | 53.69 |
| Age | ≤20 | 13835 | 67.15 |
|  | >20 | 6767 | 32.85 |
| Major | Medicine | 7835 | 38.03 |
|  | Science and technology | 6729 | 32.66 |
|  | literature, arts, or management | 4947 | 24.01 |
|  | Vocational education | 1091 | 5.30 |
| Grade | Freshman | 10339 | 50.18 |
|  | Sophomore | 4679 | 22.71 |
|  | Junior | 2532 | 12.29 |
|  | Senior | 974 | 4.73 |
|  | Graduate student | 2078 | 10.09 |
| **Knowledge accesses** |  |  |  |
| School education | Yes | 17949 | 87.12 |
|  | No | 2653 | 12.88 |
| Mass communication | Yes | 19030 | 92.37 |
|  | No | 1572 | 7.63 |
| Interpersonal communication | Yes | 11274 | 54.72 |
|  | No | 9328 | 45.28 |
| **Knowledge Scores** |  |  |  |
| Behavioral guidance knowledge | 0 point | 233 | 1.13 |
|  | 1 point | 686 | 3.33 |
|  | 2 points | 2625 | 12.74 |
|  | 3 points | 7098 | 34.45 |
|  | 4 points | 9960 | 48.35 |
| Fundamental knowledge | 0 point | 262 | 1.27 |
|  | 1 point | 220 | 1.07 |
|  | 2 points | 524 | 2.54 |
|  | 3 points | 3603 | 17.49 |
|  | 4 points | 15993 | 77.63 |
| Total score of knowledge | 0 point | 125 | 0.61 |
|  | 1 point | 104 | 0.50 |
|  | 2 points | 124 | 0.60 |
|  | 3 points | 186 | 0.90 |
|  | 4 points | 414 | 2.01 |
|  | 5 points | 1148 | 5.57 |
|  | 6 points | 3148 | 15.28 |
|  | 7 points | 6899 | 33.49 |
|  | 8 points | 8454 | 41.03 |
| **Sexual behaviors** |  |  |  |
| Having sexual intercourse? | Yes | 2423 | 11.76 |
|  | No | 18179 | 88.24 |
| Having multiple sexual partners? | Yes | 363 | 1.76 |
|  | No | 20239 | 98.24 |
| Having unprotected sex? | Yes | 830 | 4.03 |
|  | No | 19772 | 95.97 |
| Risky behaviors | Yes | 19635 | 95.31 |
|  | No | 967 | 4.69 |

**Table S2.** Path coefficient estimates of school education on behavioral guidance knowledge, fundamental knowledge and risky behaviors.^a^

| **Dependent**  **Variable** | **Independent Variable** | **B** | **SE** | **P** | **95% CI**  **(LLCI)** | **95% CI**  **(ULCI)** |
| --- | --- | --- | --- | --- | --- | --- |
|  |  |  |  |  |  |  |
| **Behavioral guidance knowledge** | School education | 0.11 | 0.01 | <.01 | 0.08 | 0.14 |
| **Fundamental knowledge** | School education | 0.15 | 0.02 | <.01 | 0.11 | 0.18 |
| **Risky behaviors** | School education | -0.23 | 0.09 | <.01 | -0.40 | -0.06 |
|  | Behavioral guidance knowledge | -0.22 | 0.05 | <.01 | -0.31 | -0.13 |
|  | Fundamental knowledge | 0.15 | 0.04 | <.01 | 0.07 | 0.24 |

^a^Note: Regression models with behavioral guidance knowledge and fundamental knowledge as dependent variables used OLS regression, with coefficients reported as unstandardized regression coefficients (B) and test statistics as t values. Regression models with risky behaviors as the dependent variable used logistic regression, with coefficients reported as unstandardized log-odds coefficients (B) and test statistics as z values. The model incorporates covariates such as age, gender, grade and major. For the sake of simplicity, the coefficients of the covariates are not reported.

**Table S3.** Direct and indirect effects of school education on risky behaviors via behavioral guidance knowledge and fundamental knowledge.^a^

| **Effect Type** | **B** | **SE** | **95% CI (LLCI)** | **95% CI (ULCI)** | **Result** |
| --- | --- | --- | --- | --- | --- |
|  |  |  |  |  |  |
| **Direct Effect** | -0.23 | 0.09 | -0.40 | -0.06 | Significantly negative |
| **Indirect effect**  **(via Behavioral guidance knowledge)** | -0.02 | 0.01 | -0.04 | -0.01 | Significantly negative |
| **Indirect effect**  **(via Fundamental knowledge)** | 0.02 | 0.01 | 0.01 | 0.04 | Significantly positive |
| **Total Indirect Effect** | 0 | 0.01 | -0.01 | 0.01 | Not significant |
| **Contrast of Indirect Effects**  **(Behavioral guidance knowledge - Fundamental knowledge)** | -0.05 | 0.01 | -0.07 | -0.03 | Significantly negative |

^a^Note: All effects are reported in a log-odds metric.

LLCI = lower limit confidence interval, ULCI = upper limit confidence interval. Bootstrap samples = 5000.

**Table S4.** Path coefficient estimates of mass communication on behavioral guidance knowledge, fundamental knowledge, and risky behaviors.^a^

| **Dependent Variable** | **Independent Variable** | **B** | **SE** | **P** | **95% CI (LLCI)** | **95% CI (ULCI)** |
| --- | --- | --- | --- | --- | --- | --- |
|  |  |  |  |  |  |  |
| **Behavioral guidance knowledge** | Mass communication | 0.2 | 0.02 | ＜.01 | 0.17 | 0.24 |
| **Fundamental knowledge** | Mass communication | 0.08 | 0.02 | ＜.01 | 0.04 | 0.13 |
| **Risky behaviors** | Mass communication | -0.24 | 0.11 | .03 | -0.46 | -0.03 |
|  | Behavioral guidance knowledge | -0.22 | 0.05 | ＜.01 | -0.31 | -0.13 |
|  | Fundamental knowledge | 0.15 | 0.04 | ＜.01 | 0.07 | 0.23 |

^a^Note: Regression models with behavioral guidance knowledge and fundamental knowledge as dependent variables used OLS regression, with coefficients reported as unstandardized regression coefficients (B) and test statistics as t values. Regression models with risky behaviors as the dependent variable used logistic regression, with coefficients reported as unstandardized log-odds coefficients (B) and test statistics as z values. The model incorporates covariates such as age, gender, grade and major. For the sake of simplicity, the coefficients of the covariates are not reported.

**Table S5.** Direct and indirect effects of mass communication on risky behaviors via behavioral guidance knowledge and fundamental knowledge.^a^

| **Effect Type** | **B** | **SE** | **95% CI (LLCI)** | **95% CI (ULCI)** | **Result** |
| --- | --- | --- | --- | --- | --- |
|  |  |  |  |  |  |
| **Direct Effect** | -0.25 | 0.11 | -0.46 | -0.03 | Significantly negative |
| **Indirect effect**  **(via Behavioral guidance knowledge)** | -0.04 | 0.01 | -0.07 | -0.02 | Significantly negative |
| **Indirect effect**  **(via Fundamental knowledge)** | 0.01 | 0.01 | 0 | 0.03 | Significantly positive |
| **Total Indirect Effect** | -0.03 | 0.01 | -0.05 | -0.01 | Significantly negative |
| **Contrast of Indirect Effects (Behavioral guidance knowledge - Fundamental knowledge)** | -0.06 | 0.02 | -0.09 | -0.03 | Significantly negative |

^a^Note: All effects are reported in a log-odds metric.

LLCI = lower limit confidence interval, ULCI = upper limit confidence interval. Bootstrap samples = 5000.

**Table S6.** Path coefficient estimates of interpersonal communication on behavioral guidance knowledge, fundamental knowledge and risky behaviors.^a^

| **Dependent Variable** | **Independent Variable** | **B** | **SE** | **P** | **95% CI**  **(LLCI)** | **95% CI**  **(ULCI)** |
| --- | --- | --- | --- | --- | --- | --- |
|  |  |  |  |  |  |  |
| **Behavioral guidance knowledge** | Interpersonal communication | −0.03 | 0.01 | <.01 | -0.05 | -0.01 |
| **Fundamental knowledge** | Interpersonal communication | 0.03 | 0.01 | .01 | 0.01 | 0.06 |
| **Risky behaviors** | Interpersonal communication | 0.22 | 0.07 | <.01 | 0.08 | 0.35 |
|  | Behavioral guidance knowledge | −0.22 | 0.05 | <.01 | -0.31 | -0.13 |
|  | Fundamental knowledge | 0.15 | 0.04 | <.01 | 0.07 | 0.23 |

^a^Note: Regression models with behavioral guidance knowledge and fundamental knowledge as dependent variables used OLS regression, with coefficients reported as unstandardized regression coefficients (B) and test statistics as t values. Regression models with risky behaviors as the dependent variable used logistic regression, with coefficients reported as unstandardized log-odds coefficients (B) and test statistics as z values. The model incorporates covariates such as age, gender, grade and major. For the sake of simplicity, the coefficients of the covariates are not reported.

**Table S7.** Direct and indirect effects of interpersonal communication on risky behaviors via behavioral guidance knowledge and fundamental knowledge.^a^

| **Effect Type** | **B** | **SE** | **95% CI (LLCI)** | **95% CI (ULCI)** | **Result** |
| --- | --- | --- | --- | --- | --- |
|  |  |  |  |  |  |
| **Direct Effect** | 0.22 | 0.07 | 0.08 | 0.35 | Significantly positive |
| **Indirect effect**  **(via Behavioral guidance knowledge)** | 0.01 | 0 | 0 | 0.01 | Significantly positive |
| **Indirect effect**  **(via Fundamental knowledge)** | 0.01 | 0 | 0 | 0.01 | Significantly positive |
| **Total Indirect Effect** | 0.01 | 0 | 0.01 | 0.02 | Significantly positive |
| **Contrast of Indirect Effects**  **(Behavioral guidance knowledge - Fundamental knowledge)** | 0 | 0 | -0.01 | 0.01 | Not significant |

^a^Note: All effects are reported in a log-odds metric.

LLCI = lower limit confidence interval, ULCI = upper limit confidence interval. Bootstrap samples = 5000.
